# A 12-gene immune signature predicts prognosis and identifies KRT6B as a therapeutic target in lung adenocarcinoma

**DOI:** 10.3389/fimmu.2026.1693469

**Published:** 2026-02-25

**Authors:** Weiwei Gu, Yahua Wu, Rongqi Jiang, Mingliang Shi, Jiude Qi, Jinhuo Lai

**Affiliations:** 1 Department of Medical Oncology, Fujian Medical University Union Hospital, Fuzhou, Fujian, China; 2 Department of Oncology, People’s Hospital Affiliated to Shandong First Medical University, Jinan, Shandong, China

**Keywords:** biomarker, immune-related gene signature, KRT6b, lung adenocarcinoma, prognostic model

## Abstract

**Background:**

Lung adenocarcinoma (LUAD) exhibits high mortality and heterogeneity. While immune-related signatures show prognostic potential, robust models validated through both computational screening and experimental methods are lacking.

**Methods:**

Transcriptomic and clinical data from The Cancer Genome Atlas (TCGA) database and three Gene Expression Omnibus (GEO) cohorts (GSE3141, GSE30219, and GSE50081) were analyzed. A 12-gene immune-related prognostic signature was constructed using LASSO Cox regression. The model was subsequently validated using three independent external cohorts. Its prognostic performance was comprehensively assessed using time-dependent receiver operating characteristic (ROC) curves. Functional enrichment analyses (GO, KEGG, and GSEA), tumor microenvironment (TME) profiling (via CIBERSORT and ESTIMATE algorithms), and drug sensitivity analyses were conducted. Protein-protein interaction (PPI) network analysis identified KRT6B as a central hub gene. KRT6B expression and its functional role were further validated through tissue microarray immunohistochemistry (IHC), as well as *in vitro* and *in vivo* experiments.

**Results:**

We developed a prognostic model for LUAD based on 12 immune-related genes and derived a risk score via LASSO regression. High-risk patients exhibited significantly worse overall survival compared to low-risk patients in both the training set (TCGA) and the three independent validation cohorts (GSE3141, GSE30219, and GSE50081) (all P < 0.05). Time-dependent ROC analysis confirmed the model’s predictive accuracy for 1-, 2-, and 3-year survival (AUC: 0.624–0.788). A nomogram incorporating the risk score and key clinical indicators further enhanced prognostic performance (AUC: 0.753 to 0.763). PPI network analysis pinpointed KRT6B as a core hub gene within the signature. Subsequent experimental validation confirmed the overexpression of KRT6B in LUAD tumor cells and demonstrated its tumor-promoting functions both *in vitro* and *in vivo*.

**Conclusion:**

We established and validated an immune-related gene signature for prognostic prediction and identified KRT6B as a promising prognostic biomarker and potential therapeutic target in LUAD.

## 1 Introduction

Lung adenocarcinoma (LUAD), the most common subtype of non-small cell lung cancer (NSCLC), remains a leading cause of cancer-related mortality worldwide. According to the latest global cancer statistics, there were 2.48 million new lung cancer cases in 2022, with LUAD accounting for 39% of male and 57% of female cases (1, 2). Despite advancements in targeted therapies and immunotherapies, the 5-year survival rate for LUAD remains below 20%, primarily due to late-stage diagnosis, high tumor heterogeneity, and treatment resistance (3).These challenges highlight the urgent need for innovative prognostic models to guide personalized treatment strategies.

Recent studies have highlighted the critical role of the tumor immune microenvironment (TME) in LUAD progression and treatment outcomes (4). The infiltration patterns, spatial distribution, and functional states of immune cells within the TME are closely linked to therapeutic responses and clinical prognosis. Immune-related genes (IRGs) play a pivotal role in regulating the TME, influencing processes such as immune evasion, inflammation, and tumor progression (5–7). Dysregulation of IRGs can lead to an immunosuppressive microenvironment, allowing tumor cells to evade immune surveillance and resist therapies, including immunotherapy. Additionally, IRGs mediate crosstalk between tumor and immune cells, affecting both tumor growth and treatment efficacy. As key regulators of the TME, IRGs have emerged as critical biomarkers and therapeutic targets in LUAD. Understanding their functional roles is essential for developing effective prognostic models and personalized treatment strategies.

Prognostic models that integrate clinical and molecular features have increasingly become a focal point of research (8, 9). Although several studies have attempted to construct IRG-based prognostic models for LUAD, existing models face significant limitations. These include a lack of multi-dimensional data integration and validation, insufficient characterization of the dynamic properties of the TME, and challenges in clinical translation, which collectively hinder their predictive performance and practical utility (10, 11). In recent years, advancements in high-throughput sequencing technologies and the integration of multi-omics data have shown tremendous potential in cancer research (12). Multi-gene models leveraging machine learning techniques have demonstrated significant advantages in managing high-dimensional data and enhancing predictive accuracy (13). For instance, the 70-gene MammaPrint model in breast cancer (14) has provided valuable insights and a reference framework for similar studies in LUAD. However, there remains a pressing need for robust prognostic models that comprehensively integrate clinical and molecular features in LUAD.

In this study, we systematically integrated transcriptomic and clinical data from TCGA and GEO cohorts to construct and validate an immune-related prognostic gene signature for LUAD. We employed comprehensive bioinformatics analyses combined with multi-level experimental validation to identify KRT6B as a central hub gene within the model. Further functional investigations revealed that high KRT6B expression is closely associated with poor clinical outcomes and promotes LUAD cell proliferation, migration, and invasion. Our findings provide novel insight into the molecular determinants of LUAD prognosis and propose KRT6B as a promising biomarker and potential therapeutic target.

## 2 Materials and methods

### 2.1 Data acquisition and processing

The transcriptome data and clinical materials of LUAD (n = 512) were downloaded from TCGA (https://portal.gdc.cancer.gov/). The clinical features are detailed in **Supplementary Table S1**. Moreover, we selected three independent validation datasets (GSE3141, GSE30219, and GSE50081) from the GEO database (http://www.ncbi.nlm.nih.gov/geo/) and obtained their normalized microarray gene expression and clinical data (**Supplementary Table S2**). We downloaded functional gene sets from the Molecular Signatures Database (MSigDB) and filtered these sets by applying the keyword ‘immune’ to obtain the target genes. All transcriptome and clinical data from the TCGA and GEO databases are publicly available and were collected according to their respective data access policies. The datasets contain only de-identified patient information; thus, institutional review board approval and informed consent were not required for this study.

### 2.2 Identification of prognostic IRGs

After collection and preprocessing of LUAD data, the “limma” R package (15) was employed to identify differentially expressed genes (DEGs) between tumor and normal tissues, using a fold change (FC) > 1.5 and false discovery rate (FDR) < 0.05 as thresholds. Subsequently, univariable cox regression analysis was performed on the identified genes to determine those with prognostic value (P < 0.05). Immune-related DEGs were then screened. Finally, by analyzing the intersection of these two gene sets, immune-related genes with prognostic significance were identified.

### 2.3 Construction and verification of immune-related gene signature

The least absolute shrinkage and selection operator (LASSO) regression analysis of the candidate prognostic IRGs was performed to construct a prognostic gene signature. Then, we calculated each patient’s risk score using the following formula: Risk score = ∑Coef (mRNA) × Expression (mRNA) (where Coef represents the regression coefficient). Patients in the TCGA training group were divided into high-risk and low-risk groups based on the median risk score. To validate the predictive performance of the model, three independent validation datasets (GSE3141, GSE30219, and GSE50081) were included in our study. Risk scores were calculated for each sample in both the training cohort and the validation cohorts using the same formula. Based on the median risk score, patients were divided into high-risk and low-risk subgroups to explore the prognostic differences between the two groups. Kaplan-Meier curves and receiver operating characteristic (ROC) curves were constructed for both the training cohort and the validation cohorts.

### 2.4 Independent prognostic analysis and nomogram construction

To evaluate whether the immune-related gene signature could serve as an independent prognostic factor in LUAD patients, we performed a multivariate cox regression analysis. A nomogram predicting overall survival (OS) at 1, 2, and 3 years was constructed using the “rms” R package, incorporating patient age, gender, stage, and risk scores.

### 2.5 Functional enrichment analysis

We utilized the “limma” R package to identify differentially expressed genes (DEGs) between the high-risk and the low-risk groups, with the criteria of fold change (FC) > 2 and false discovery rate (FDR) < 0.05 (16). The results of the Gene Ontology (GO) and Kyoto Encyclopedia of Genes and Genomes (KEGG) analyses were visualized using the “circlize” R package (17). Additionally, Gene Set Enrichment Analysis (GSEA) with the Kolmogorov–Smirnov (KS) test was conducted to identify enriched KEGG pathways. A ridge plot was generated to visualize the details of the GSEA results using the “ggstatsplot” R package.

### 2.6 Risk model’s association with TME

The Immuno-Oncology Biological Research (IOBR) R package (18) was used to analyze the immune features and immune cell infiltration in high- and low-risk groups. Sample enrichment scores were calculated based on TME-associated signatures provided by the IOBR package. The expression levels of these signatures were compared between the two risk groups, and their correlation with the risk score was assessed using the Pearson correlation test. The CIBERSORT algorithm in the IOBR package was employed to estimate the relative abundance of 22 immune cell types in the TCGA-LUAD cohort. Additionally, the ESTIMATE algorithm was used to calculate matrix and immune scores for each sample, offering deeper insights into the TME.

### 2.7 Drug sensitivity analysis

The Cancer Therapeutics Response Portal (CTRP) database and the Genomics of Drug Sensitivity in Cancer (GDSC) database provide data on the sensitivity of tumor cells to various anti-tumor drugs. Using the R package “oncoPredict” (19), we calculated the sensitivity of individual LUAD patients to different anti-tumor drugs based on their gene expression data (log2(TPM + 1)). The differences in area under the dose–response curve (AUC) values between the high-risk and low-risk groups were then analyzed to evaluate variations in drug sensitivity.

### 2.8 Validation of model genes by RT-qPCR

To validate the expression of the bioinformatically identified model genes at the cellular level, we selected three human lung adenocarcinoma cell lines (A549, PC9, H1975) and one human normal lung epithelial cell line (BEAS-2B) as controls. Total RNA was extracted from each cell line using the Eastep^®^ Super Total RNA Extraction Kit, and its concentration and purity were measured. Subsequently, 1 μg of total RNA was reverse-transcribed into first-strand cDNA following the instructions of the reverse transcription kit. Using the cDNA as a template, amplification was performed with SYBR Green premix reagent on a real-time quantitative PCR system. The primer sequences are listed in **Supplementary Table S3**. The housekeeping gene ACTIN was used as an internal reference, and the relative expression levels of each model gene across different cell lines were calculated using the 2^–ΔΔCt method. All experiments were conducted with three technical replicates to ensure data reliability.

### 2.9 Identification and multi-omics validation of the hub gene

Prognostic stratification was performed based on a 12-gene immune signature, and the top 200 most significantly upregulated genes in the high-risk group were subjected to protein-protein interaction (PPI) network analysis. Interaction data were obtained from the STRING database (version 12.0, https://string-db.org/) with a confidence score threshold of 0.4. The PPI network was visualized and analyzed using Cytoscape software (version 3.9.1). Hub genes within the network were identified using the cytoHubba plugin, which applied five topological analysis methods. Genes with the highest centrality scores were defined as hub genes for further analysis. KRT6B was ultimately identified as the hub gene. KRT6B expression was analyzed using the TISCH (https://tisch.compbio.cn/home/) and Cancer SCEM (https://ngdc.cncb.ac.cn/cancerscem/index) databases. Its mRNA expression and association with clinical stage in LUAD were assessed using TCGA and GEO (GSE115002 dataset), while promoter methylation levels were evaluated via UALCAN database.

### 2.10 Experimental validation

Immunohistochemistry (IHC) was performed on formalin-fixed paraffin-embedded (FFPE) LUAD tissue microarrays to evaluate the protein expression level of the hub gene KRT6B. IHC results were independently assessed by two blinded senior pathologists using a semi-quantitative scoring system (H-score based on staining intensity and percentage of positive cells). The correlation between KRT6B expression levels and patient overall survival was further analyzed. To investigate the biological function of KRT6B, human lung adenocarcinoma cell lines A549 and PC9 were selected. Stable knockdown of KRT6B was achieved using lentivirus-mediated delivery of shRNA targeting the sequence 5’-CTGAAACTCAGTCTAGGTCCACTCGAGTGGACCTAGACTGAGTTTCAG-3’. Non-targeting scramble shRNA was used as a control. Knockdown efficiency was confirmed by RT-qPCR and Western blotting. For *in vitro* functional assays, cell proliferation was measured using the CCK-8 and colony formation assays. Cell invasion and migration capacities were assessed using Transwell and wound healing assays, respectively. For *in vivo* studies, control and KRT6B-knockdown A549 cells were subcutaneously injected into BALB/c nude mice (n = 5 per group). Tumor volume was measured twice weekly starting from day 7 post-injection using a digital caliper, and monitoring continued for 28 days. At the endpoint, tumors were excised and weighed. KRT6B and Ki67 expression in tumor tissues were detected by immunohistochemistry, and co-expression of major immune cells within the tumor microenvironment was analyzed by multiplex immunofluorescence.

### 2.11 Statistical analysis

R software version 4.3.3 was used to conduct the statistical analysis, and the p-value below 0.05 was regarded as statistically significant.

## 3 Results

### 3.1 Immune-related genes with significant prognostic value in LUAD

**Figure 1:**
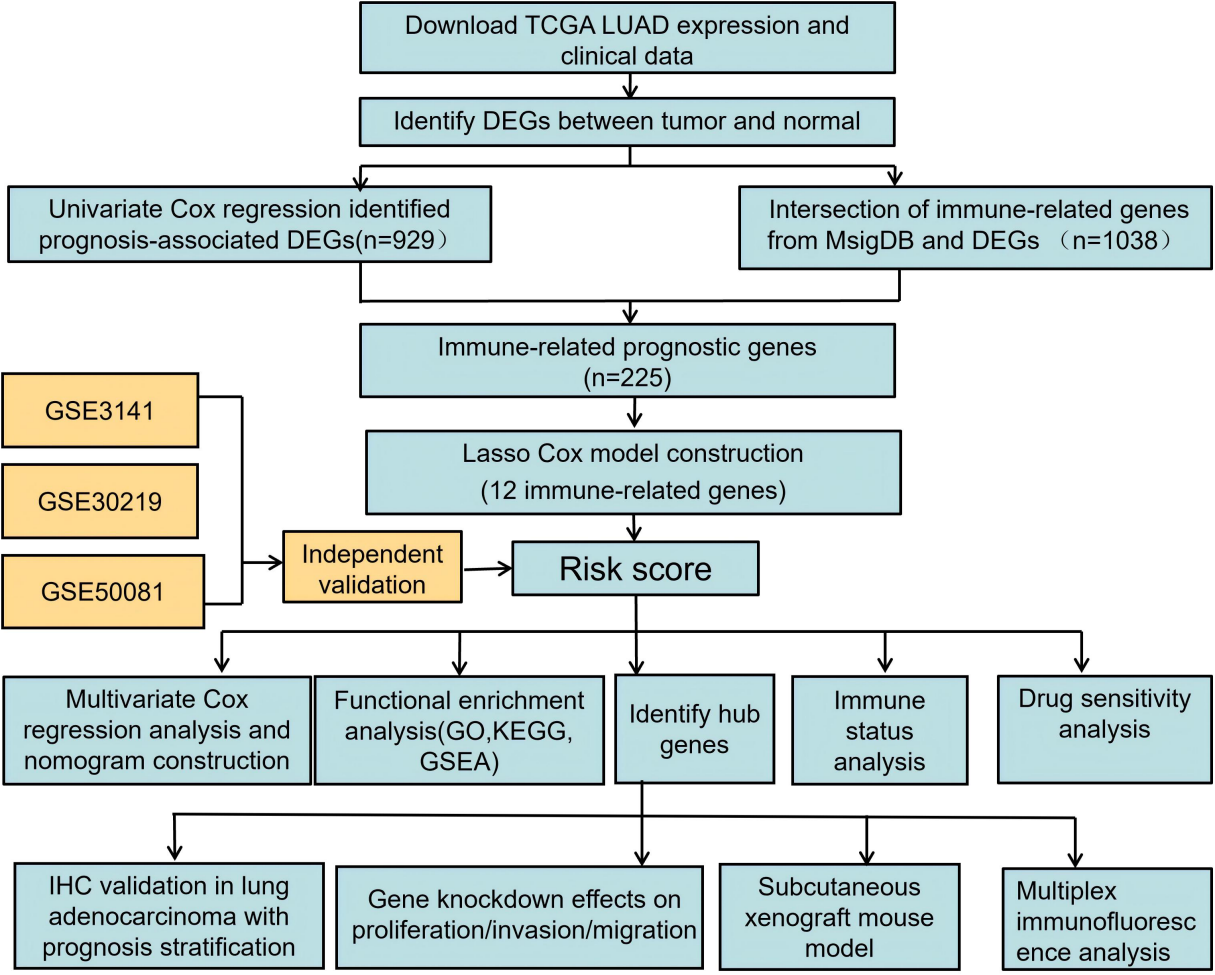
Flow chart of the study.

The study design is illustrated in the flow chart (**Figure 1**). The clinical baseline characteristics of lung adenocarcinoma patients are provided in **Supplementary Table S1**.

### 3.2 Establishment and validation of an immune-related gene signature model

**Figure 2:**
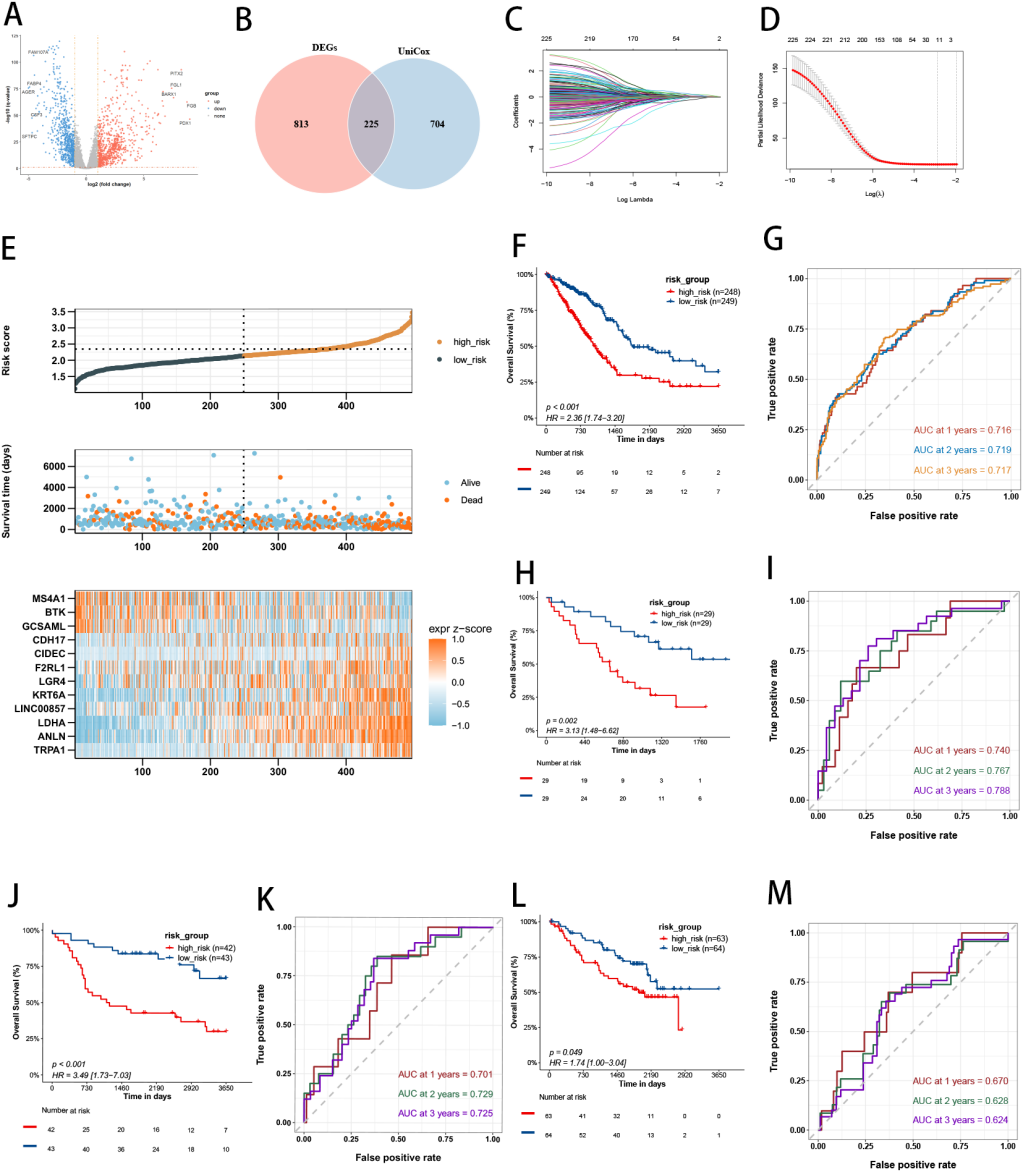
Dentification of Prognostic IRGs and Development of a Risk Model. **(A)** Differential expression of immune-related genes in tumor versus normal tissues. **(B)** Intersection analysis of immune-related differentially expressed genes and prognosis-associated genes. **(C)** LASSO coefficient profiles of 12 prognosis-related IRGs; different colors represent different genes. **(D)** LASSO regression with tenfold cross-validation and selection of the optimal parameter (lambda) in the LASSO model. **(E)** The distribution of risk scores, survival status, and heatmap of LUAD patients in the TCGA cohort. **(F)** Kaplan-Meier survival curves for overall survival (OS) between low-risk and high-risk groups in the TCGA cohort. **(G)** Time-dependent ROC curves for 1-, 2-, and 3-year survival of LUAD patients in the TCGA cohort. **(H)** Kaplan-Meier survival curves for OS between low-risk and high-risk groups in the GSE3141 cohort. **(I)** Time-dependent ROC curves for 1-, 2-, and 3-year survival of LUAD patients in the GSE3141 cohort. **(J)** Kaplan-Meier survival curves for OS between low-risk and high-risk groups in the GSE30219 cohort. **(K)** Time-dependent ROC curves for 1-, 2-, and 3-year survival of LUAD patients in the GSE30219 cohort. **(L)** Kaplan-Meier survival curves for OS between low-risk and high-risk groups in the GSE50081cohort. **(M)** Time-dependent ROC curves for 1-, 2-, and 3-year survival of LUAD patients in the GSE50081 cohort.

The expression of several immune-related genes exhibits significant differences between tumor tissues and normal tissues (**Figure 2A**). As shown in **Figure 2B**, a total of 929 immune-related DEGs were identified. Additionally, 1,038 immune-related prognostic genes were determined using univariate cox regression analysis. Intersection analysis revealed 225 immune-related prognostic differentially expressed genes (IRGs), which were subsequently subjected to LASSO regression analysis to identify key genes (**Figures 2C, D**).

We constructed a prognostic model consisting of 12 IRGs using LASSO regression analysis. These genes include LDHA, TRPA1, GCSAML, ANLN, CIDEC, KRT6A, LGR4, F2RL1, BTK, MS4A1, LINC00857, and CDH17. The risk score was calculated as follows: Risk score = (0.18434 × LDHA expression) + (0.134316 × TRPA1 expression) + (-0.12171 × GCSAML expression) + (0.083437 × ANLN expression) + (0.077786 × CIDEC expression) + (0.040304 × KRT6A expression) + (0.014487 × LGR4 expression) + (0.013599 × F2RL1 expression) + (-0.01291 × BTK expression) + (-0.01195 × MS4A1 expression) + (0.007756 × LINC00857 expression) + (0.002833 × CDH17 expression).

Each LUAD patient’s risk score was calculated based on LASSO coefficients and gene expression values. Risk scores, survival status, and heatmaps in the TCGA cohort were analyzed (**Figure 2E**). Risk curves and scatter plots showed that higher risk scores were associated with higher mortality. Kaplan-Meier analysis revealed that low-risk patients had significantly better survival than high-risk patients (P < 0.001, **Figure 2F**). The AUCs of ROC curves for 1-, 2-, and 3-year survival were 0.716, 0.719, and 0.717, respectively (**Figure 2G**), indicating the model’s effectiveness in predicting LUAD prognosis.

To validate the stability and generalizability of our model, the GSE3141, GSE30219 and GSE50081 cohorts were used as external validation datasets, The clinical baseline characteristics of lung adenocarcinoma patients are provided in **Supplementary Table S2**. Kaplan-Meier curves showed significantly better survival in the low-risk group compared to the high-risk group in three cohorts (P < 0.05, **Figures 2H, J, L**).The ROC curves demonstrated the model’s predictive accuracy for 1-, 2-, and 3-year survival. In the GSE3141 cohort, the AUCs were 0.740, 0.767, and 0.788, respectively (**Figure 2I**). In the GSE30219 cohort, the AUCs were 0.701, 0.729, and 0.725, respectively (**Figure 2K**). In the GSE50081 cohort, the AUCs were 0.670, 0.628, and 0.624, respectively (**Figure 2M**).These results confirm the model’s strong predictive performance.

### 3.3 Creation of nomograms based on IRGs signatures combined with clinical characteristics

**Figure 3:**
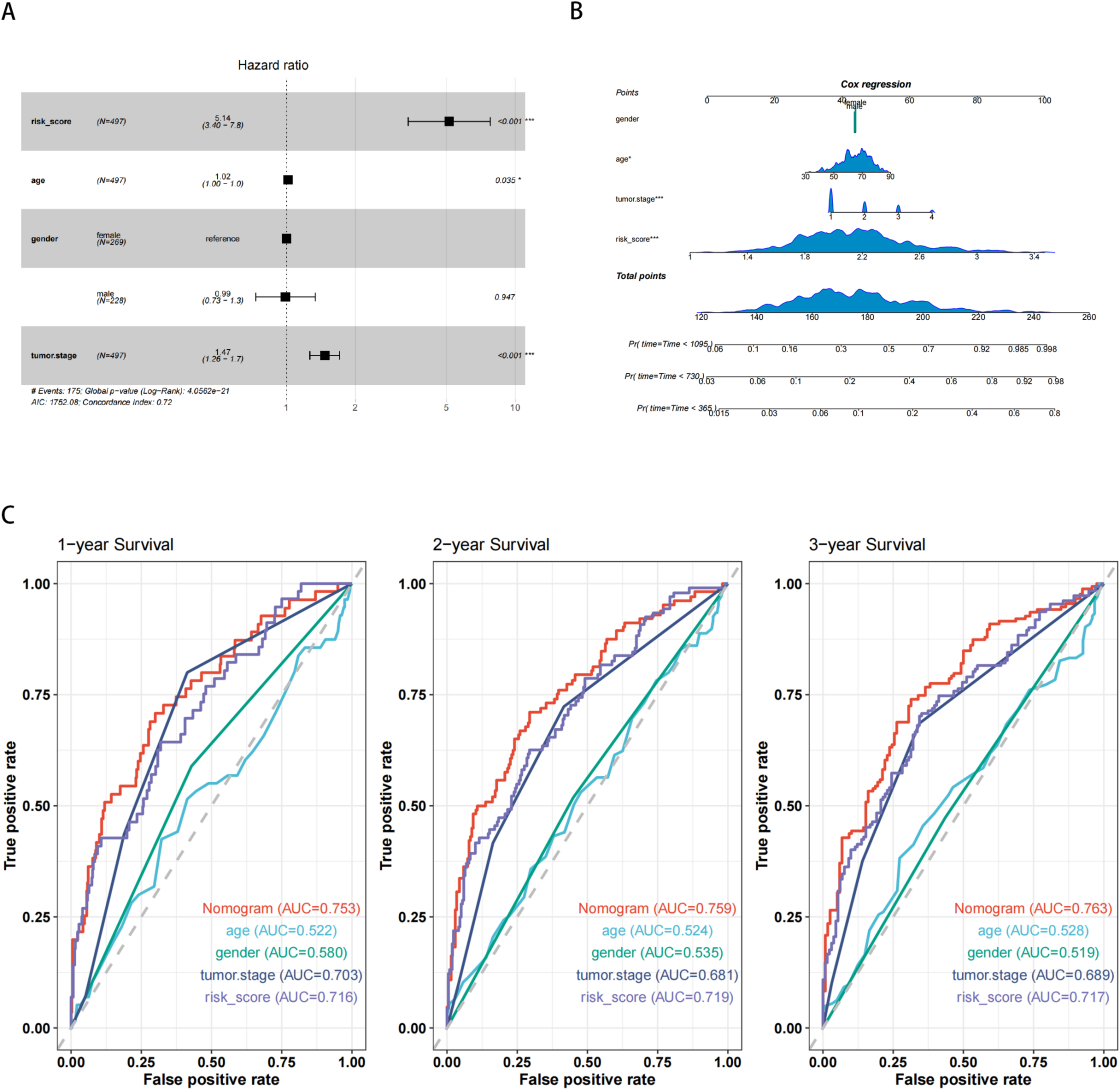
Construction and validation of the nomogram model. **(A)** Multivariate Cox analysis indicated that the risk score was an independent prognostic factor significantly associated with overall survival (OS) in the TCGA cohort. **(B)** Nomogram for predicting 1-year, 2year, and 3-year OS based on the TCGA cohort. **(C)** Time-dependent ROC curve analysis comparing the predictive performance of the nomogram, risk score, age, gender, and tumor stage in the TCGA cohort. ***P < 0.001 indicates statistical significance.

To validate the reliability and clinical value of the biological signature constructed based on IRGs as a predictor of prognosis, we conducted multivariate cox regression analysis. The results showed that tumor stage (P < 0.001) and risk scores (P < 0.001) were independent prognostic factors significantly associated with patients outcomes (**Figure 3A**). To quantitatively predict patients’ prognosis and inform clinical decision-making, we integrated the risk scores with clinical indicators to construct nomograms for predicting 1-year, 2-year, and 3-year survival probabilities (**Figure 3B**). Time-dependent ROC analysis was then performed to compare the predictive accuracy of the nomogram, risk scores, and other common clinical features (**Figure 3C**). The results indicated that the risk scores had a much higher AUC compared to individual clinical features such as tumor stage and age. Furthermore, the nomogram demonstrated superior prognostic accuracy, with higher AUC values than the risk scores alone. The time-dependent AUCs of the nomogram for predicting 1-year, 2-year, and 3-year overall survival (OS) were 0.753, 0.759, and 0.763, respectively.

### 3.4 Identification of DEGs between high-risk and low-risk groups and function enrichment analysis

**Figure 4:**
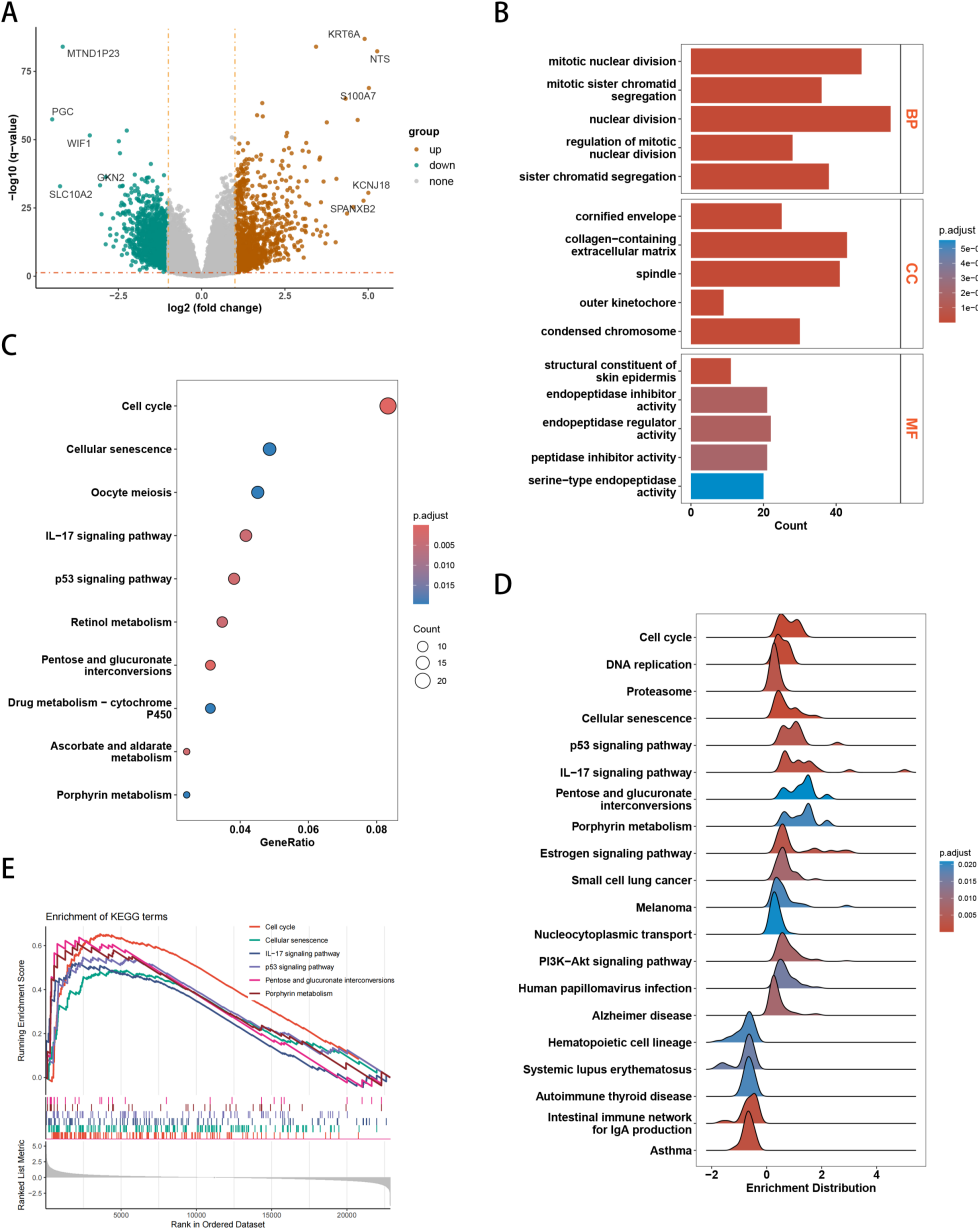
Results of differentially expressed genes (DEGs) and functional enrichment. **(A)** Volcano plot of DEGs between high-risk and low-risk groups. **(B)** GO enrichment analysis of DEGs. **(C)** KEGG enrichment analysis of DEGs. **(D)** Ridge plot of KEGG pathways identified by GSEA. **(E)** GSEA plot of key pathways between the two groups.

Differentially expressed genes (DEGs) were analyzed in the TCGA cohort for high-and low-risk groups, with results shown in **Figure 4A**. Gene enrichment analysis of upregulated genes in the high-risk group revealed significant findings. Gene Ontology (GO) analysis showed enrichment in cell division-related processes (e.g., sister chromatid segregation and mitotic nuclear division regulation) under the biological process (BP) category, chromosomal structures and extracellular matrix-related components under the cellular component (CC) category, and enzyme activity regulation and epidermal structure formation under the molecular function (MF) category (**Figure 4B**). KEGG pathway analysis indicated significant enrichment in pathways such as cell cycle (highest gene ratio), cellular senescence, oocyte meiosis, IL-17 signaling, and p53 signaling, as well as metabolism-related pathways, including retinol metabolism, pentose and glucuronate interconversions, cytochrome P450-mediated drug metabolism, ascorbate and aldarate metabolism, and porphyrin metabolism (**Figure 4C**). GSEA further identified enriched pathways in high-risk samples, including cell cycle, DNA replication, proteasome, cellular senescence, p53 signaling, IL-17 signaling, metabolic pathways (pentose and glucuronate interconversions, porphyrin metabolism), and disease-related pathways such as melanoma, systemic lupus erythematosus, and autoimmune thyroid disease (**Figure 4D**). Both KEGG and GSEA analyses consistently highlighted significant enrichment in cell cycle, cellular senescence, IL-17 signaling, p53 signaling, and metabolism-related pathways (**Figure 4E**).

### 3.5 Immune signatures between high-risk and low-risk groups

**Figure 5:**
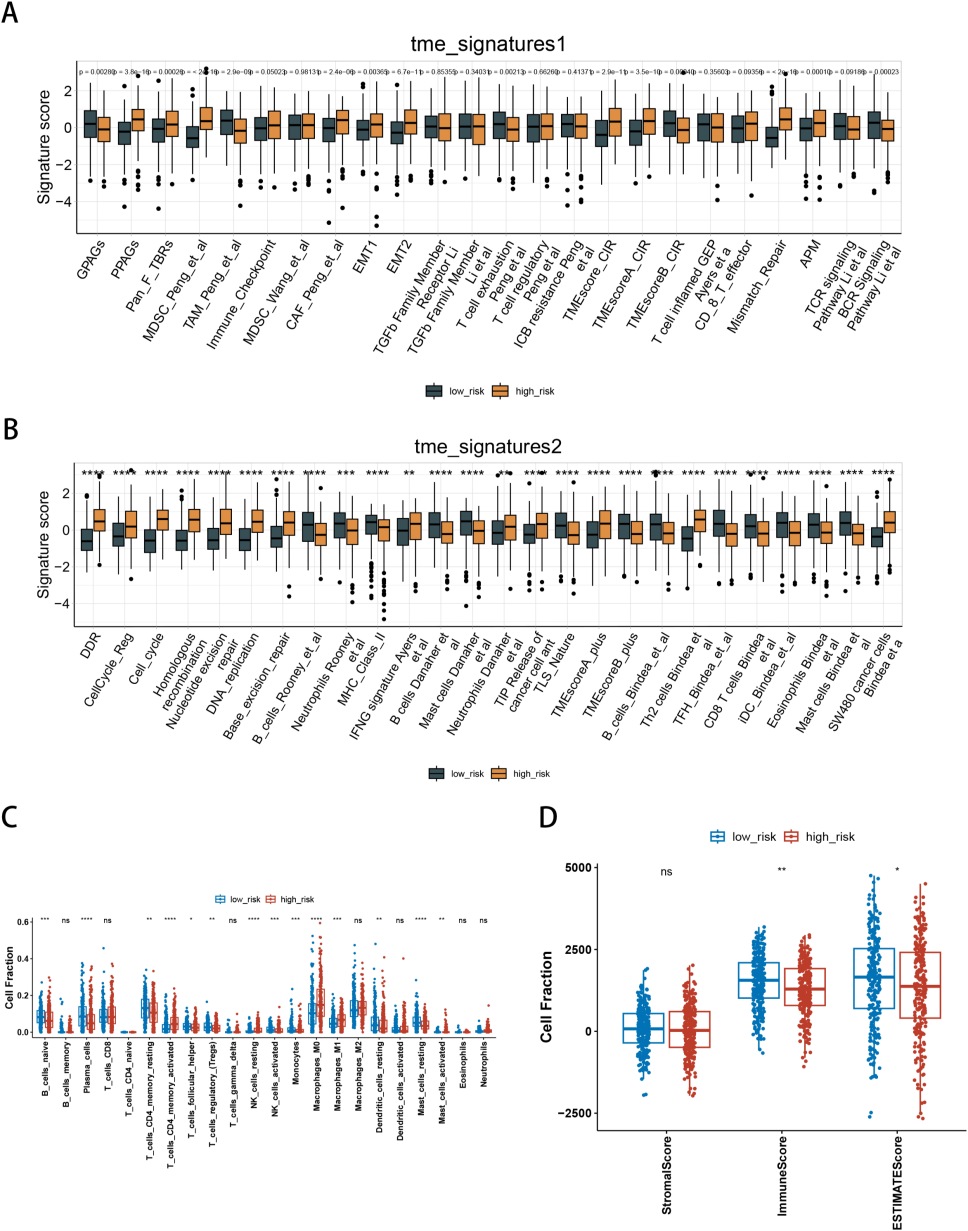
Immune signatures between high-risk and low-risk groups. **(A, B)** Tumor microenvironment (TME)-associated signatures; **(C)** Proportions of immune cell components assessed by CIBERSORT in the TCGA cohort; **(D)** Differences in ESTIMATE immune infiltration scores (stromal score, immune score, and ESTIMATE score) between high-risk and low-risk groups in the TCGA cohort. *P < 0.05, **P < 0.01, ***P < 0.001, ****P < 0.0001, ns, not significant.

To further elucidate differences in the immune microenvironment of patients between high-risk and low-risk groups, we compared the enrichment scores of TME signatures between the two groups. In **Figure 5A**, the high-risk group demonstrated higher expression of PPAGs, Pan_F_TBRs, MDSC_Peng_et_al, CAF_Peng_et_al, EMT1, EMT2, TMEScore_CIR, TMEScoreA_CIR, APM, and the TCR signaling pathway. Conversely, the low-risk group exhibited higher expression of GPAGs, TAM_Peng_et_al, T cell exhaustion, TMEScoreB_CIR, and the BCR signatures. In **Figure 5B**, the high-risk group exhibited elevated expression of several features, such as DNA damage response, cell cycle regulation, homologous recombination, nucleotide excision, DNA replication, base excision repair, IFNG signature, TIP release of cancer cell antigens, TME score A+, Th2 Tcells, and SW480 cancer cells. In contrast, the low-risk group is characterized by high expression of features including B cells, neutrophils, MHC class II, additional B cells, TLS nature, TME score B+, CD8 T cells, iDC, eosinophils, TFH, and mast cells. We estimated the proportion of 22 types of immune cells in each sample by CIBERSORT algorithm. **Figure 5C** shows the differences in the proportions of immune cell types between the two risk groups. The high-risk group exhibited elevated levels of activated CD4 memory T cells, resting NK cells, M0 macrophages, and M1 macrophages. Conversely, the low-risk group demonstrated elevated levels of naive B cells, plasma cells, resting CD4 memory T cells, follicular helper T cells, regulatory T cells (Tregs), activated NK cells, monocytes, and both regular and M1 dendritic cells. The results of ESTIMATE suggested that immune score and ESTIMATE score were higher in the low-risk group (**Figure 5D**).

### 3.6 Relationship between risk scores and response to drugs

**Figure 6:**
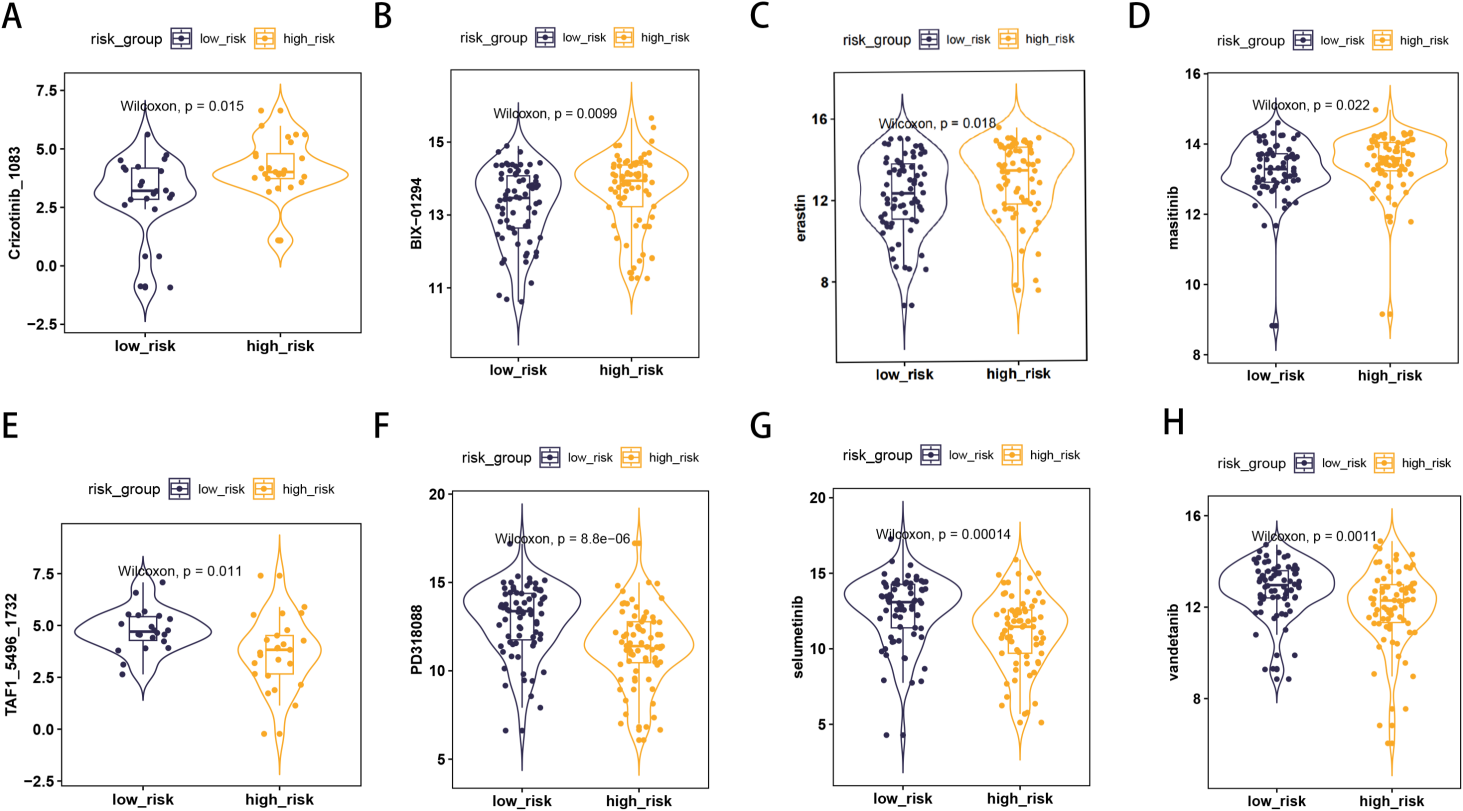
Risk score model predicts the sensitivity of anti-tumor drugs. **(A)** Crizotinib_1083; **(B)** BIX_01294; **(C)** erastin; **(D)** masitinib; **(E)** TAF1_5496_1732; **(F)** PD318088; **(G)** selumetinib; **(H)** vandetanib.

To identify more effective drugs for patients in the high-risk group, we analyzed differences in tumor cell sensitivity to chemotherapeutic drugs between risk groups based on the GDSC and CTRP databases (**Figure 6**). We found that patients in the high-risk group tended to be less sensitive to Crizotinib_1083, BIX_01294, erastin, and masitinib, but more sensitive to TAF1_5496_1732, PD318088, selumetinib, and vandetanib.

### 3.7 Validation of twelve signature prognostic genes by RT-qPCR

**Figure 7:**
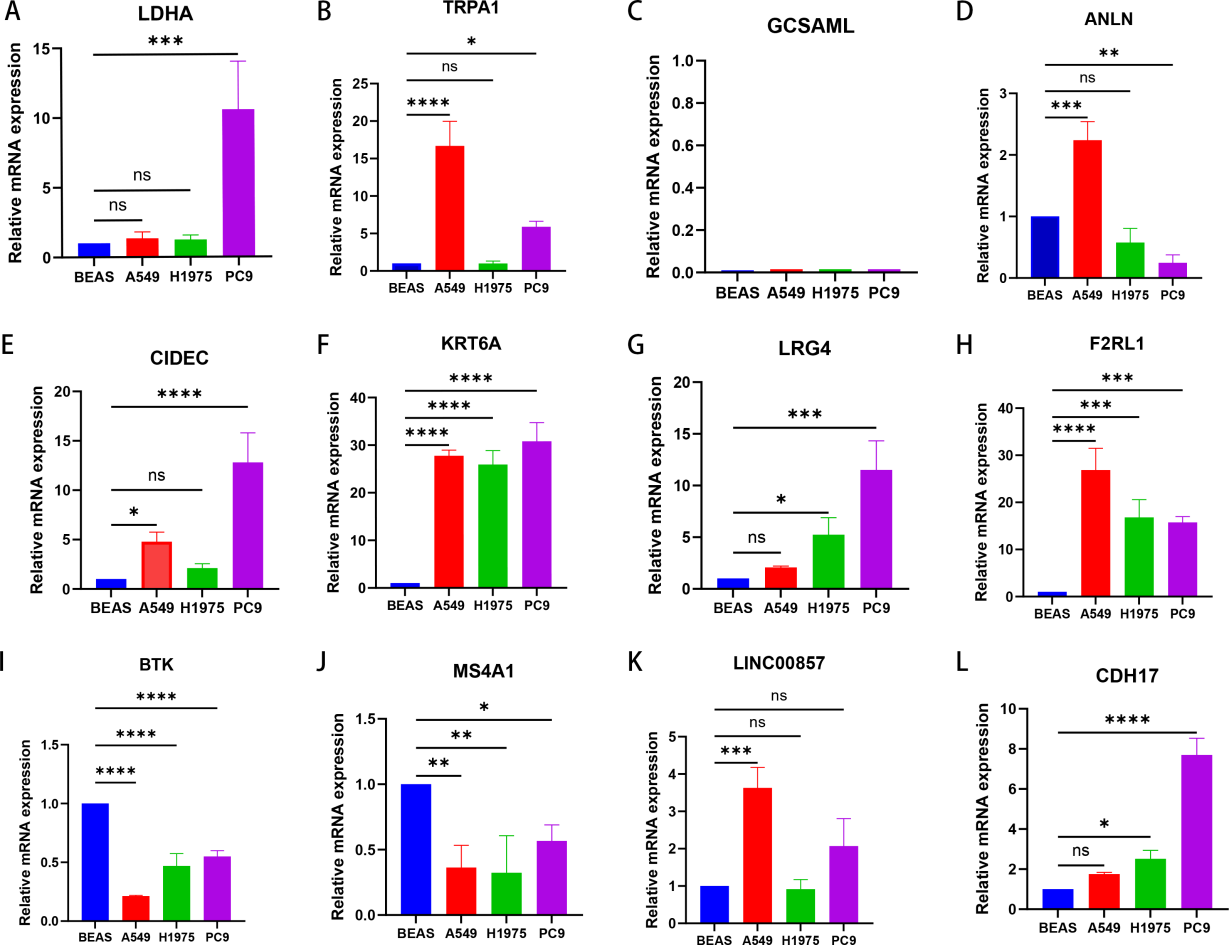
Evaluation of the expression of twelve immune-related signature genes in normal and LUAD cells. LDHA **(A)**, TRPA1 **(B)**, GCSAML **(C)**, ANLN **(D)**, CIDEC **(E)**, KRT6A **(F)**, LGR4 **(G)**, F2RL1 **(H)**, BTK **(I)**, MS4A11 **(J)**, LINC00857 **(K)**, and CDH17 **(L)**. *P < 0.05, **P < 0.01, ***P < 0.001, ****P < 0.0001; ns, not significant.

To further verify the twelve signature genes in normal lung epithelial cells and LUAD cell lines, quantitative real-time polymerase chain reaction (RT-qPCR) was performed (**Figures 7A–L**). The results showed that in LUAD cell lines, the expression of LDHA, TRPA1, ANLN, CIDEC, KRT6A, LGR4, F2RL1, LINC00857, and CDH17 was elevated, whereas the expression of BTK and MS4A11 was decreased. GCSAML expression was not detected in BEAS-2B human normal lung epithelial cell line or the three human LUAD cell lines. The expression levels of the signature genes in clinical samples were further validated through immunohistochemistry data obtained from the HPA database, with detailed results presented in **Supplementary Figure 1**.

### 3.8 Stratification of LUAD patient prognosis using an immune-related gene signature and identification of hub genes

**Figure 8:**
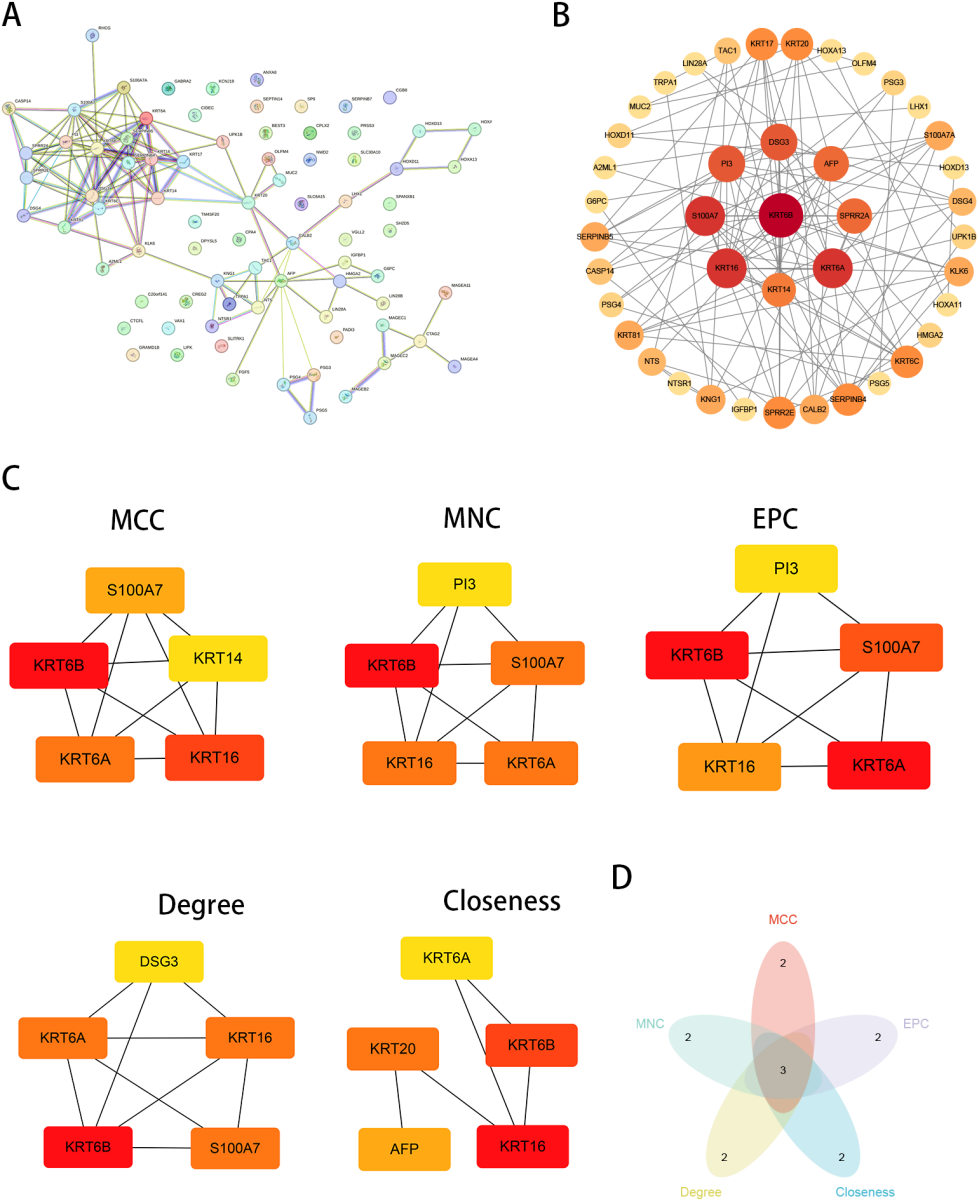
Dentification of Hub Genes via PPI Network Analysis. **(A)** PPI Network Analysis of Top 200 Upregulated Genes from High-Risk Subgroup Using STRING Database. **(B)** Protein–protein interaction (PPI) network analysis of Cytoscape. **(C)** five topological algorithms. **(D)** Intersection analysis of these algorithm-specific gene sets revealed three consensus hub genes.

To identify pivotal prognostic hub genes, we extracted the top 200 upregulated genes from the high-risk subgroup and constructed a Protein-Protein Interaction (PPI) network using the STRING database (**Figure 8A**) This network was subsequently analyzed in Cytoscape (**Figure 8B**) with five topological algorithms (MCC, MNC, EPC, etc.) (**Figure 8C**), from which the top five highest-ranked genes per algorithm were selected. Intersection analysis of these algorithm-specific gene sets revealed three consensus hub genes: KRT6B, KRT16, and KRT6A (**Figure 8D**). Among these, KRT6B demonstrated the highest composite ranking score and was designated as the central hub gene.

### 3.9 Single-cell level analysis of KRT6B in the LUAD TME

**Figure 9:**
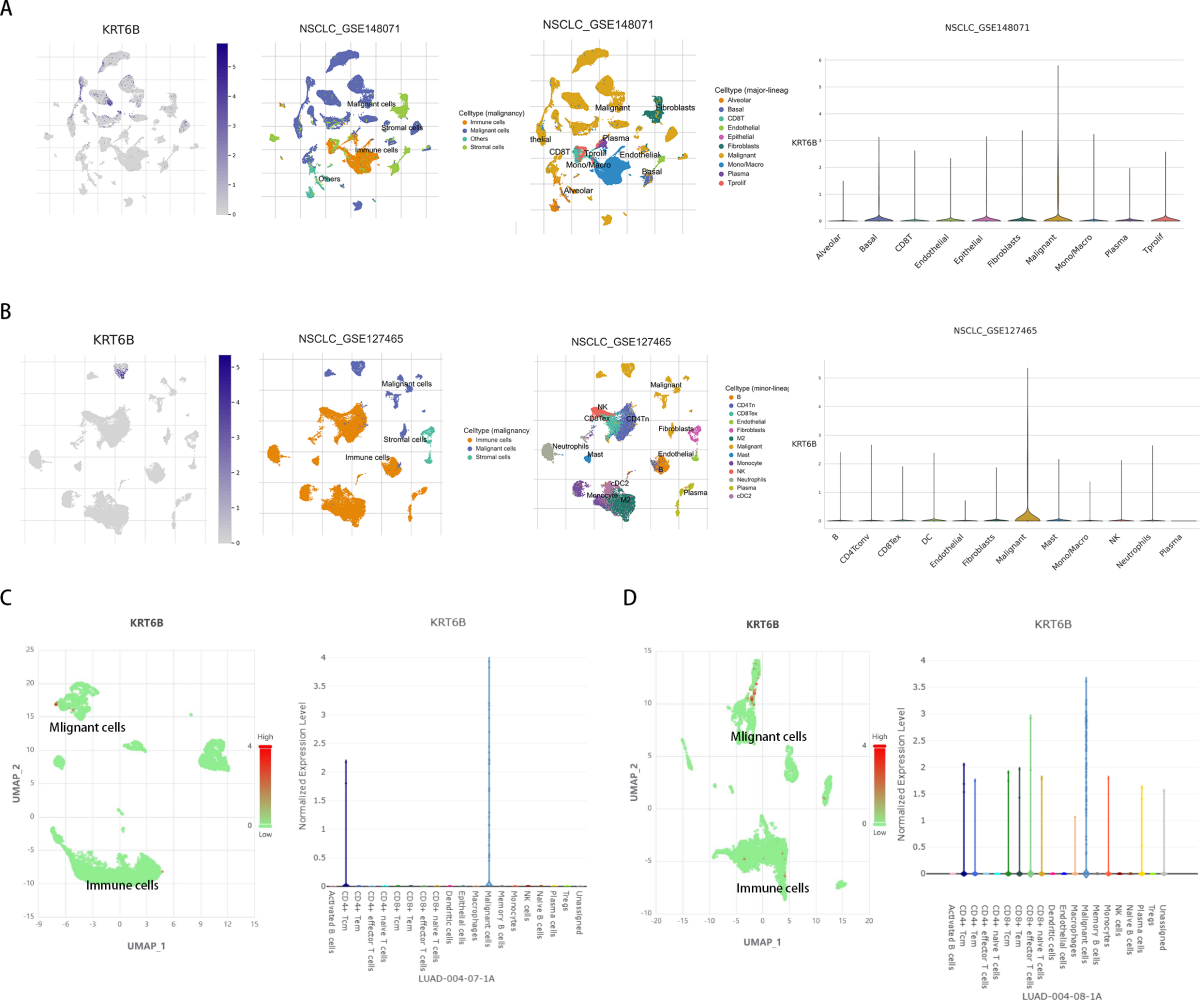
Expression of KRT6B at single-cell resolution. **(A)** Cell-type-stratified visualization in the NSCLC_GSE148071 dataset. **(B)** Cell-type-stratified visualization in the NSCLC_GSE127465 dataset. **(C, D)** Cell-type-stratified visualization in LUAD.

We analyzed the expression profile of KRT6B in the non-small cell lung cancer (NSCLC) tumor microenvironment (TME) at single-cell resolution using datasets (GSE1148071 and GSE127465) from the Tumor Immune Single-cell Hub (TISCH) database. Results demonstrated broad expression of KRT6B across malignant cells and various immune cell populations, with the highest expression levels observed in tumor cells (**Figures 9A, B**). Subsequent validation using single-cell expression data from the Cancer SCEM database for LUAD patients similarly indicated that KRT6B is predominantly expressed in malignant cells (**Figures 9C, D**). Collectively, these findings suggest that KRT6B may have potential functional implications within the TME of LUAD.

### 3.10 Transcriptomic prognostic value of KRT6B with IHC-level verification

**Figure 10:**
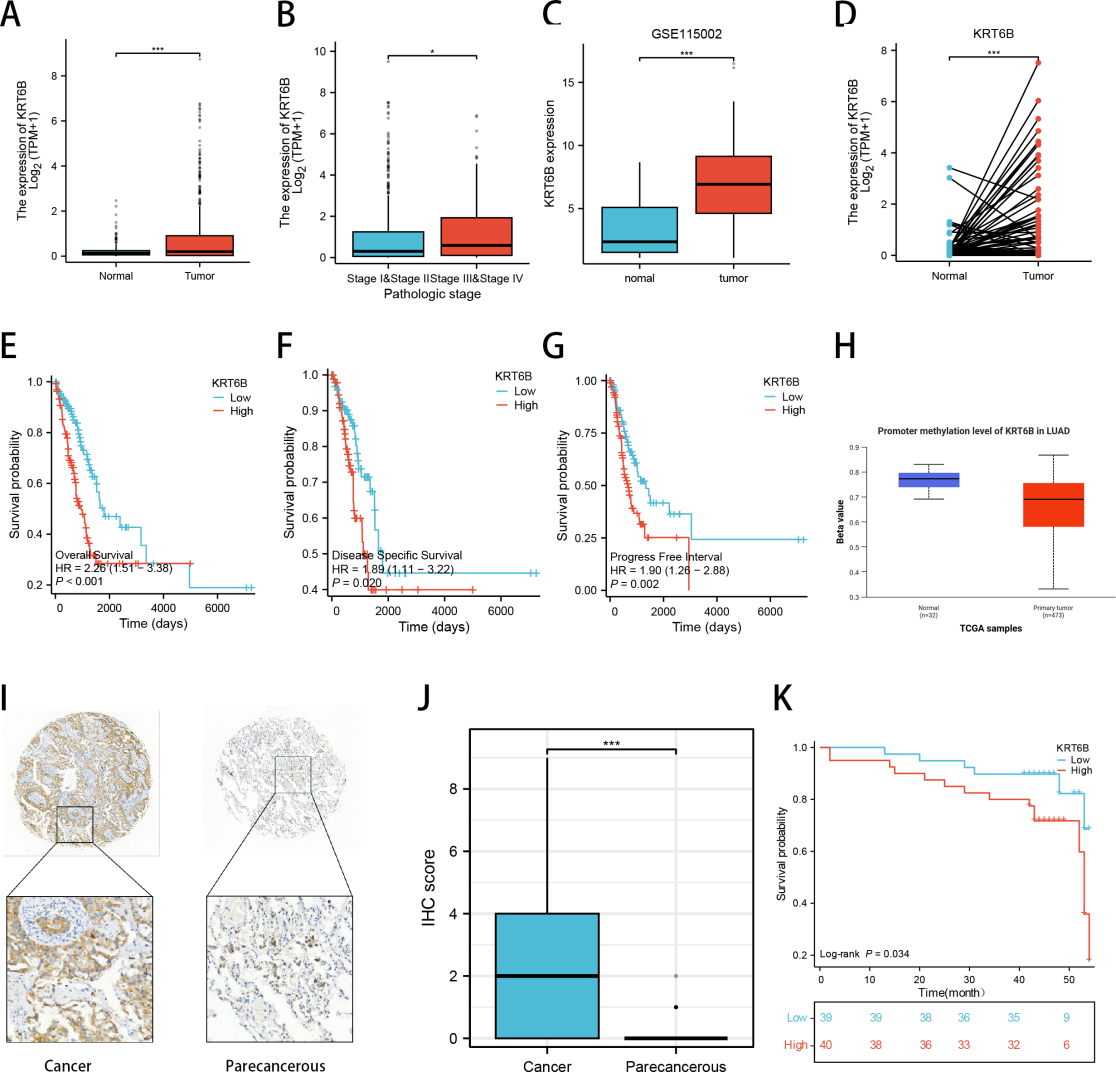
Comprehensive evaluation of KRT6B’s expression and prognostic significance in LUAD. **(A–D)** Transcriptomic analysis from the TCGA and GEO databases confirms KRT6B mRNA is significantly overexpressed in LUAD tissues compared to normal controls, and its expression correlates with advanced clinical stage. **(E–G)** Survival analysis demonstrates that high KRT6B mRNA expression is associated with significantly poorer overall survival (OS), disease-specific survival (DSS), and progression-free interval (PFI). **(H)** Epigenetic analysis via UALCAN reveals hypomethylation of the KRT6B promoter in LUAD, suggesting a potential mechanism for its upregulation. **(I–K)** Protein-level validation using a LUAD tissue microarray (n=79 matched pairs). **(I)** Representative immunohistochemical (IHC) images of KRT6B expression in LUAD and paracancerous tissues. **(J)** Quantitative analysis confirms significantly higher KRT6B protein levels in tumor tissues. **(K)** Kaplan-Meier analysis verifies that high KRT6B protein expression is a prognostic marker for worse overall survival. *P < 0.05, ***P < 0.001.

Evaluation of KRT6B mRNA expression levels in TCGA database revealed significant overexpression in LUAD (**Figure 10A**), with differential expression patterns across clinical stages (**Figure 10B**). This finding was independently validated in the GSE115002 dataset, where KRT6B mRNA levels were consistently elevated in LUAD samples (**Figure 10C**). Concordantly, paired-sample analysis in TCGA demonstrated significantly higher KRT6B expression in tumor tissues compared to adjacent normal controls (**Figure 10D**). Survival analysis across multiple endpoints revealed that high KRT6B expression was significantly associated with poorer overall survival (OS), disease-specific survival (DSS), and progression-free interval (PFI) (**Figures 10E–G**). Furthermore, UALCAN database analysis showed significantly reduced promoter methylation levels of KRT6B in LUAD versus normal lung tissue (**Figure 10H**). KRT6B protein levels were assessed using the LUAD tissue microarray. Following exclusion of specimens with inadequate cores or missing data, 79 matched pairs of tumor and adjacent normal tissues were included for subsequent analysis. Representative immunohistochemical images demonstrating differential KRT6B expression in LUAD versus paracancerous tissues are presented in **Figure 10I**. Quantitative analysis revealed significantly higher immunohistochemical staining scores in LUAD tissues compared to matched normal counterparts (**Figure 10J**). Survival analysis further indicated that patients with high KRT6B expression exhibited worse overall survival outcomes than those with low expression (P = 0.034; **Figure 10K**).

### 3.11 Verifying KRT6B function *in vivo* and *in vitro*

**Figure 11:**
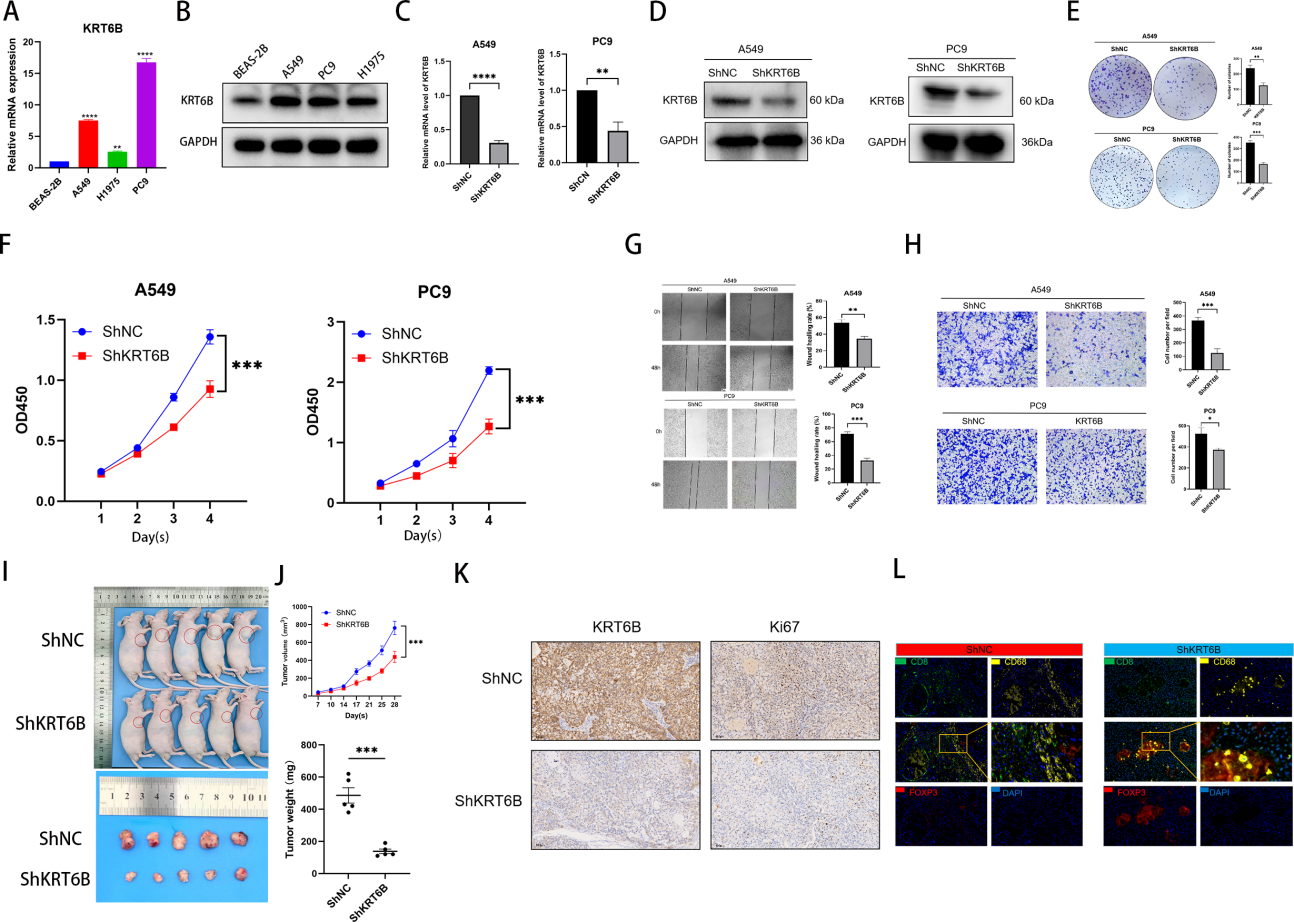
**(A)** Expression levels of mRNA **(B)** Expression levels of protein **(C)** Changes in KRT6B mRNA expression after knockdown of KRT6B. **(D)** Changes in KRT6B protein expression after knockdown of KRT6B. **(E)** Impact of KRT6B on the proliferation of lung adenocarcinoma cells examined by plate **(F)** CCK8 assay to test the effect of KRT6B on the proliferation of lung adenocarcinoma cells **(G)** The effect of KRT6B on the migratory ability of LUAD cells was examined by cell **(H)** The effects of KRT6B on the invasion capacity of LUAD cells were examined by transwell invasion assays. **(I)** Gross images of tumor formation in control and knockdown nude mice. **(J)** Statistical plots of tumor volume and weight in control versus knockdown nude mice. **(K)** Immunohistochemical assay was used to detect the expression of KRT6B and Ki67 in tumor tissues of nude mice in control and knockdown groups. **(L)** Multiplex Immunofluorescence Histochemistry for Comparative Analysis of the Tumor Immune Microenvironment in Knockdown versus Control Groups. *P < 0.05, **P < 0.01, ***P < 0.001, ****P < 0.0001.

The RT-qPCR and Western blotting analysis revealed significant up-regulation of KRT6B in LUAD cell lines (A549, PC9, H1975) compared to normal human lung epithelial BEAS-2B cells (**Figures 11A, B**). To functionally characterize KRT6B in LUAD pathogenesis, we established stable knockdown models in A549 and PC9 cells (**Figures 11C, D**). Phenotypic assessment demonstrated that KRT6B depletion significantly attenuated malignant behaviors: colony formation assays revealed impaired proliferative capacity (**Figure 11E**), CCK-8 assays confirmed growth inhibition (**Figure 11F**), cell wound healing assay showed reduced migration potential (**Figure 11G**), and transwell assays indicated suppressed invasion capability (**Figure 11H**). *In vivo* assay demonstrated that KRT6B knockdown significantly inhibited the proliferative capacity of LUAD cells in nude mouse xenograft models (**Figure 11I**). Subcutaneous implantation of KRT6B-knockdown A549cells resulted in markedly reduced tumor growth compared to control groups, as evidenced by both decreased tumor volume and lower tumor weight (**Figure 11J**). The Ki-67 protein is an important marker associated with cell proliferation. We conducted an immunosuppression experiment by transplanting tumors from nude mice. As shown in the figure, the expression of the Ki-67 protein in the transplanted tumor tissue of the knockdown group was significantly lower than that in the control group (**Figure 11K**), suggesting that silencing KRT6B *in vivo* can inhibit the proliferation of lung cancer cells. To further investigate changes in the immune microenvironment of xenograft tumors, we performed multiplex immunofluorescence to spatially localize CD8^+^ T cells, macrophages, and regulatory T cells. The results revealed that compared with the control group, the KRT6B knockdown group exhibited significantly reduced infiltration of CD8^+^ T cells and macrophages, along with a relative increase in regulatory T cell infiltration within the transplanted tumors (**Figure 11L**). This pattern aligns with the immune-suppressive microenvironment characteristics observed between the high-risk and low-risk groups in our prognostic model, further supporting the hypothesis that KRT6B may contribute to lung adenocarcinoma progression by modulating the tumor immune microenvironment.

## 4 Discussion

Lung adenocarcinoma (LUAD), the predominant histologic subtype of non-small-cell lung cancer, is characterized by marked inter- and intra-tumoral heterogeneity, resulting in highly variable therapeutic responses and clinical outcomes. Current prognostication relies predominantly on the TNM classification, which provides limited granularity for precision risk stratification (20). For instance, even patients with the same stage may exhibit significant differences in genomic features, immune microenvironment status, and treatment responses. Therefore, the development of molecular feature-based prognostic models has become a critical direction in LUAD research (1). Advances in multi-omics sequencing and bioinformatics have systematically elucidated the key genes and pathways that drive LUAD progression and prognosis (21). Driver genes such as EGFR, KRAS, and ALK influence tumor growth, metastasis, and responses to targeted therapies (22), while immune-related genes like PD-L1, CTLA-4, and TIM-3 significantly impact immunotherapy efficacy and patient survival (23). Leveraging multi-omics data and clinical information, researchers have developed prognostic models using public databases like TCGA and GEO, improving prediction accuracy and providing critical guidance for personalized treatment strategies (24).

This study integrated transcriptomic data from The Cancer Genome Atlas (TCGA) and three independent Gene Expression Omnibus (GEO) cohorts to construct a refined 12-gene immune-related prognostic signature. In contrast to previously reported models that often incorporate dozens to hundreds of genes, our signature retains robust predictive accuracy (1–3 year AUC: 0.624–0.788) while substantially reducing analytical complexity. More importantly, we identified KRT6B as a central hub gene within this signature. Although the pro-tumorigenic roles of certain keratin family members have been established in other cancer types (25–27), the specific function and clinical relevance of KRT6B in LUAD, particularly its role in modulating the tumor immune microenvironment remain poorly understood. To our knowledge, this study is the first to comprehensively establish KRT6B as a pivotal hub gene within an immune-related prognostic signature and to provide functional experimental validation of its tumor-promoting effects in LUAD. Elevated KRT6B protein levels are significantly associated with poorer overall survival. Functional assays further show that genetic silencing of KRT6B impairs proliferation, migration, and invasion of LUAD cells *in vitro*, and markedly restrains tumor growth in murine xenograft models, providing direct evidence for KRT6B as a potential therapeutic target. From a translational perspective, we integrated the risk score with TNM stage and other clinical parameters to build a nomogram that improved 1–3-year survival prediction (AUC: 0.753–0.763) compared with traditional staging alone.

Pathway enrichment analysis revealed significant differences in biological pathways between high-risk and low-risk groups. Key molecular mechanisms in the high-risk group included dysregulation of the cell cycle, activation of inflammatory signaling, and metabolic reprogramming. The enrichment of cell cycle and cellular senescence pathways indicated a higher proliferative capacity and immune evasion in tumor cells (28), while abnormalities in IL-17 and p53 signaling pathways highlighted dysregulation of the inflammatory microenvironment and tumor suppressor functions (29, 30). This suggests that KRT6B may drive disease progression through bidirectional crosstalk between tumor cells and the immune microenvironment. Additionally, the enrichment of pathways such as pentose and glucuronate interconversions and porphyrin metabolism in the high-risk group reflected enhanced metabolic adaptability of tumor cells, which is closely associated with rapid tumor growth and invasiveness (31). Single-cell analyses confirm that KRT6B is predominantly expressed in malignant cells, supporting a tumor-cell-autonomous mechanism that remodels the microenvironment (32). The concurrent up-regulation of metabolic pathways (pentose and glucuronate interconversions, porphyrin metabolism) implies that KRT6B may also enhance biosynthetic capacity and antioxidant potential, thereby accelerating tumor growth. These findings provide potential therapeutic targets for personalized treatment in LUAD, such as CDK inhibitors, anti-inflammatory therapies, and metabolic-targeted drugs.

Significant differences in tumor microenvironment (TME) characteristics were observed between the low-risk and high-risk groups. The high-risk group exhibited higher scores in immunosuppressive features, such as MDSC and TMEscoreA, indicating a more active immunosuppressive mechanism that may inhibit effector immune cell functions and promote tumor immune evasion (33, 34). Additionally, elevated EMT1 and EMT2 scores in the high-risk group suggest greater tumor invasiveness and metastatic potential (35), while higher scores in DNA damage repair (DDR) and cell cycle pathways reflect increased genomic instability and proliferative capacity (36). In contrast, the low-risk group showed a significantly higher TAM score, suggesting that tumor-associated macrophages (TAMs) in this group may play a more immunoregulatory role rather than solely contributing to immunosuppression (37). Furthermore, the low-risk group exhibited higher ImmuneScore, and ESTIMATEScore, along with an increased proportion of regulatory T cells (Tregs), indicating stronger immune activation, enhanced immune regulation, and the ability to maintain immune balance while suppressing excessive inflammation (38). Strikingly, KRT6B is predominantly expressed in malignant epithelial cells, and its high level correlates positively with the immunosuppressive signature of the high-risk group and negatively with immune-cell infiltration. Further multiplex immunofluorescence analysis in this study revealed that KRT6B knockdown not only directly inhibited tumor cell proliferation but also significantly reshaped the immune microenvironment in xenograft tumors. Specifically, it led to a marked reduction in the infiltration density of CD8^+^ T cells and macrophages, accompanied by a relative increase in the proportion of regulatory T cells (Tregs). These experimental findings align closely with our transcriptome-based risk stratification model, in which the high-risk group similarly exhibited an immunosuppressive microenvironment characterized by relative enrichment of Tregs and functional impairment of effector immune cells. Notably, although KRT6B knockdown partially alleviated the overall immunosuppressive state, a residual elevation in Treg proportions persisted, suggesting that KRT6B may play a pivotal bridging role in connecting the intrinsic malignant phenotype of tumor epithelial cells with immune microenvironment dysregulation. Its high expression likely promotes immune evasion through a dual mechanism: actively recruiting or inducing immunosuppressive cell populations while simultaneously suppressing the infiltration and function of effector immune cells. In summary, the integrated data from this study indicate that KRT6B systematically coordinates the formation of an immunosuppressive tumor microenvironment by recruiting myeloid-derived suppressor cells (MDSCs), inducing epithelial-mesenchymal transition (EMT), and activating the MEK-ERK signaling pathway. This effectively couples the intrinsic oncogenic programs of tumor cells with immune evasion mechanisms. As a central molecular node linking epithelial plasticity to microenvironmental immune dysfunction, KRT6B provides a critical theoretical foundation for developing personalized therapeutic strategies targeting the tumor microenvironment in lung adenocarcinoma.

Drug resistance remains one of the leading causes of mortality in cancer patients. Using the CTRP and GDSC databases, we explored the potential relationship between drug sensitivity and immune-related gene risk scores (39). The results demonstrated that patients in the high-risk group exhibited lower sensitivity to certain drugs, such as Crizotinib and BIX_01294, but higher sensitivity to others, such as Selumetinib and Vandetanib. These differences may be attributed to specific gene expression patterns and pathway activation in the high-risk group. For instance, resistance to Crizotinib may be associated with abnormal activation of the ALK signaling pathway or other resistance mechanisms in this group. Conversely, patients in the high-risk group showed higher sensitivity to certain drugs, such as TAF1_5496_1732 and PD318088, suggesting that these drugs may be more effective in tumors with a highly active immune microenvironment. These findings provide valuable insights for personalized treatment strategies. Specifically, patients in the high-risk group may benefit more from drugs targeting cell cycle or metabolic pathways, while those in the low-risk group could respond better to immunotherapy or drugs targeting specific signaling pathways.

This study has several limitations that should be acknowledged. The model was developed and validated using retrospective public data, and the absence of detailed clinical treatment information in some validation cohorts limits its integration into clinical decision-making. Although standardization and expression consistency analyses (e.g., Z-score distribution, Spearman correlation) were applied to mitigate technical differences between RNA-seq and microarray platforms, cross-dataset batch effect correction was not performed, which could be addressed in future studies with more homogeneous data. Functionally, experiments were conducted primarily in classical mutant cell lines (A549, PC9) and have not yet encompassed other key driver-gene subtypes of LUAD such as ALK or RET fusions. Moreover, while KRT6B appears to influence the tumor immune microenvironment, its precise molecular mechanisms remain to be elucidated. Finally, the prognostic model and the biomarker potential of KRT6B require prospective clinical validation, and the drug-sensitivity predictions derived from public databases need experimental confirmation. Future work should therefore focus on prospective cohort studies, mechanistic exploration in broader preclinical models, and the development of standardized assays to advance clinical translation.

## 5 Conclusions

We integrated transcriptomic and functional analyses to establish a 12-gene immune-related signature in which KRT6B functions as a central hub. The signature robustly stratifies LUAD patients into distinct risk groups, outperforming conventional TNM staging alone, and links KRT6B-mediated tumor–immune crosstalk. These findings provide a clinically actionable framework for refining prognosis and guiding targeted or immunotherapeutic strategies in LUAD.

## Data Availability

Publicly available datasets were analyzed in this study. This data can be found here: The datasets analyzed during the current study are available from the corresponding author on reasonable request.
