# Supplementary figures and images for: A 12-gene immune signature predicts prognosis and identifies KRT6B as a therapeutic target in lung adenocarcinoma
Source: Front Immunol. 2026 Feb 25;17:1693469. doi: 10.3389/fimmu.2026.1693469 (PMC12975743; doi:10.3389/fimmu.2026.1693469)

## KRT6B in BEAS-2B/A549/PC9/H1975

KRT6B

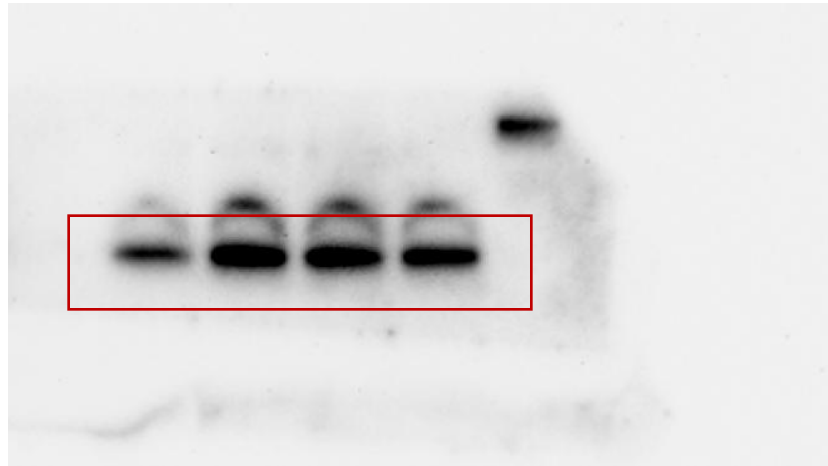

GAPDH

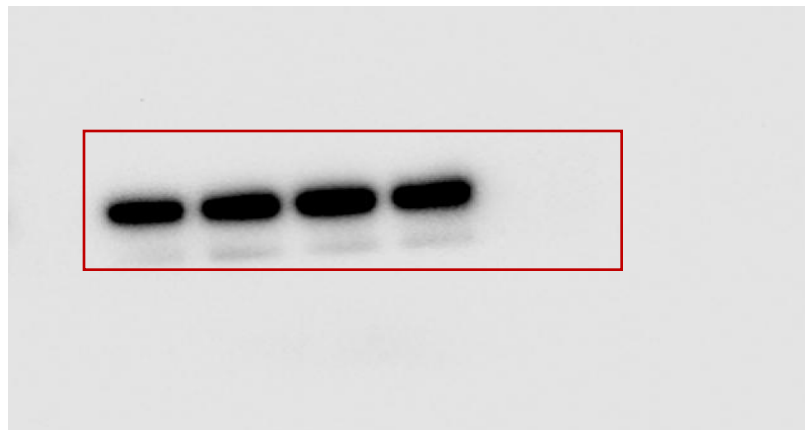

A549      ShNC and ShKRT6B

KRT6B

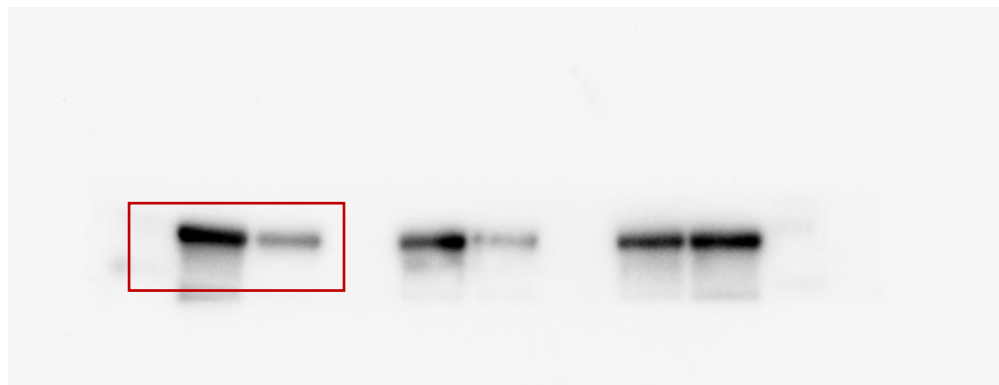

GAPDH

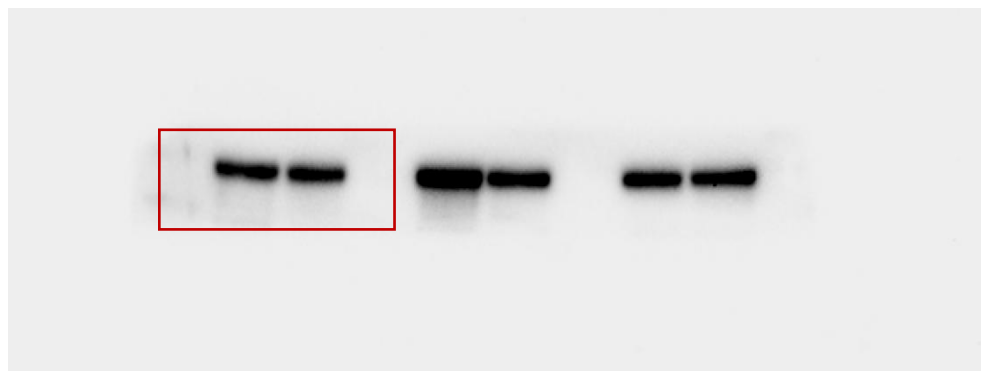

PC9 ShNC and ShKRT6B

KRT6B

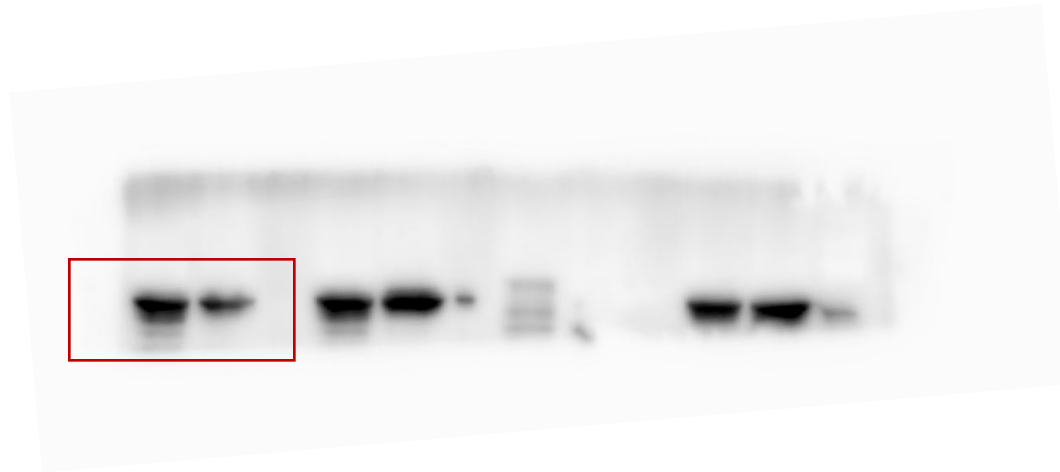

GAPDH

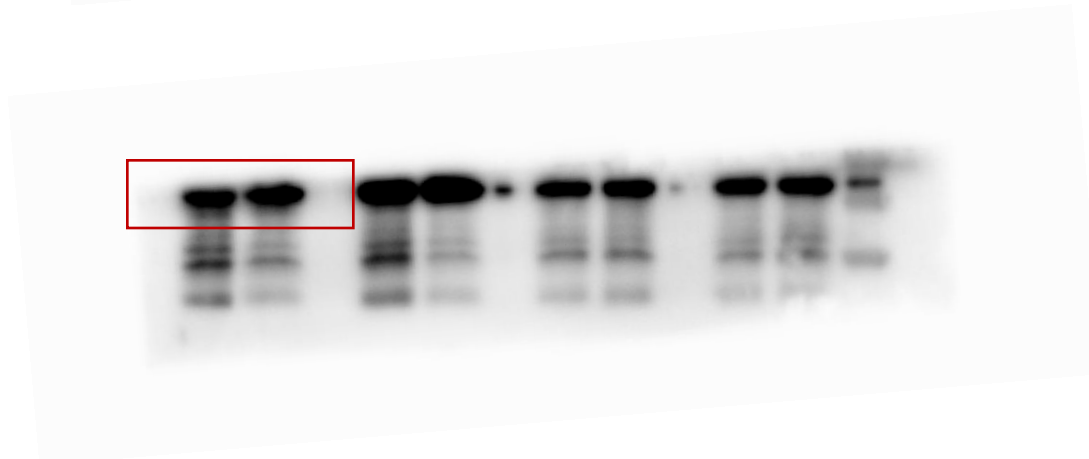

Supplement: Supplementary file 1 [file DataSheet1.pdf]
